# Antennal sensilla and brain morphology during development in caddisflies

**DOI:** 10.1007/s00441-026-04085-2

**Published:** 2026-06-19

**Authors:** Silvana Piersanti, Manuela Rebora, Gianandrea Salerno, Sylvia Anton

**Affiliations:** 1 Dipartimento Di Chimica, Biologia E Biotecnologie, University of Perugia, Via Elce Di Sotto 8, 06123 Perugia, Italy; 2 Dipartimento Di Scienze Agrarie, Alimentari E Ambientali, University of Perugia, Borgo XX Giugno 74, 06121 Perugia, Italy; 3 IGEPP, INRAE, Institut Agro, University of Rennes, Institut Agro, 2, Rue André Le Nôtre 49045, Angers Cedex 01, France

**Keywords:** Aquatic insects, Trichoptera, Ultrastructure, Neuroanatomy, Mechanoreception

## Abstract

The present study investigates the antennae and brain of the immature stages of the caddisfly *Hydropsyche pellucidula* (Andersen, and Klubnes 1834) using scanning and transmission electron microscopy as well as fluorescence microscopy. The larval antenna is unsegmented and bears two long, articulated trichoid sensilla and two large, non-articulated basiconic sensilla, all with an internal structure typical of mechanoreceptors. No changes were detected between the larval stages examined, but larval sensilla differ completely from the adult sensilla previously described in the same species, which include several chemoreceptors (e.g. trichoid, pseudoplacoid, pseudoplacoid, chaetoid, coronary, and styloconic sensilla). A preliminary account of larval brain anatomy reveals a small central body without columnar elements, mushroom bodies without calyces, and no antennal lobes in any larval stage. This condition differs markedly from the pupal brain, which—similar to the adult brain—shows a central body with a vague fan-shaped structure, mushroom bodies with small calyces, and well-structured antennal lobes containing few but relatively large glomeruli. Such dramatic change is similar to what occurs in other closely related holometabolous insects, such as Lepidoptera, but it is more pronounced, probably because of the ecological differences between larvae and adults, as also observed in other aquatic insects such as mosquitoes. The results of this research shed light on overlooked aspects of caddisfly biology. Moreover, they may enhance our understanding of the evolution of insect olfaction, since caddisflies are among the most important orders of aquatic insects and the closest relatives of Lepidoptera, a key model system in insect chemical ecology.

## Introduction

Trichoptera, commonly known as caddisflies, encompass approximately 17,000 recognized species of aquatic insects that are found globally in rivers, streams, and lakes (Del-Claro 2019; Morse 2026). These insects are well known for the intricate cases that some larvae construct as shelters. Adults, which often fly during twilight, resemble small moths with fine hairs covering their bodies and wings. The larvae are also valuable biological indicators of water quality due to their crucial role in aquatic food webs and their general sensitivity to pollution (Holzenthal et al. 2015). While significant knowledge has been produced on the taxonomy, environmental monitoring, and general zoology of these insects, there remain gaps in our understanding of key aspects of their biology, such as sensory biology and neuroanatomy (Rebora et al. 2019).

Antennae are the primary sensory organs in insects, housing various types of sensilla with different sensitivities and distributions, which are crucial for behaviour. Adult caddisflies possess conspicuous antennae that may vary in length depending on species and sex (Crespo 2011; Rebora et al. 2019). Both males and females have an incredibly wide variety of antennal sensilla, with more than 20 different types and subtypes, many of which serve chemoreceptive functions (Melnitsky and Ivanov 2011; Ivanov and Melnitsky 2011; Melnitsky et al. 2018; Abu Diiak et al. 2021). Olfaction in adult caddisflies is important for mating communication, as sex pheromone production has been documented in secretory glands located in the head and thorax of many species (Roemhild 1980; Wood and Resh 1984; Resh et al. 1987; Löfstedt et al. 1994; Löfstedt et al. 2008). Otherwise, because larvae spend their lives in rivers, we could expect a reduction in olfactory sensilla on their antennae, as well as a reduced role of olfaction in their behaviour, as is commonly reported for aquatic insects (Crespo 2011).

Trichoptera and Lepidoptera share a close phylogenetic relationship, and many similarities between the two groups are well documented (Grimaldi and Engel 2005; Zhou et al. 2016). Regarding antennal olfactory sensilla, six types have been described in both Trichoptera and Lepidoptera adults (Valuyskiy et al. 2020), but comparative analyses suggest a shift from sensilla placodea/auricillica in Trichoptera and basal moths to sensilla trichodea in derived Lepidoptera. Paralleling there is a change from Type 0 to Type I sex pheromones in derived moths (Yuvaraj 2017; Yuvaraj et al. 2018a). Transcriptomic data support this, showing ionotropic receptors in Trichoptera, whereas Pheromone Receptor clade Odorant Receptors and Pheromone-Binding proteins occur only in derived Lepidoptera, using Type I pheromones (Yuvaraj et al. 2018b; Yin et al. 2021).

Similar to Lepidoptera, larval trichopterans possess highly reduced antennae represented by one or two apical papillae (Crespo 2011; Rebora et al. 2019). However, whereas caterpillars have several antennal olfactory sensilla with different functions (Wang et al. 2025), the situation in caddisfly larvae is less clear. Many species, belonging to Rhyacophilidae and other families, lack antennal chemoreceptors (Friedrich, 2015; Crespo 2011), but a plate structure on the antenna of *Melampophylax mucoreus* (Trichoptera, Limnephilidae) has been tentatively interpreted as a multiporous plate sensillum, based on comparison with a similar structure in the larva of *Homoeosoma nebulella* (Lepidoptera, Pyralidae) (Spanhoff et al. 2005). Furthermore, the antenna of *Limnephilus centralis* has been described as equipped with olfactory and gustatory sensilla (Akent’eva 2012).

Despite extensive knowledge of Lepidoptera brain morphology and function, the general brain morphology of caddisflies has been poorly investigated (Hagberg 1986; Dacks et al. 2006; Crespo 2011; Rebora et al. 2019; Strausfeld and Olea-Rowe 2021). Recently, two PhD theses have examined the adult brain of *Rhyacophila nubila*. One compared the glomerular organization of the antennal lobe in Trichoptera with that of moths, in search of the so-called macroglomerular complex (Yuvaraj 2017). The other explored the neural basis of migration in Bogong moth and used *R. nubila* as an outgroup species (Adden 2026). To the best of our knowledge, no neuroanatomical data are available for caddisfly larvae. Nor have any studies addressed developmental changes in brain morphology from larvae to adults. This stands in contrast to the data available for Lepidoptera (Sehadova et al. 2023), as well as for other aquatic insects, namely Diptera, Odonata, and Plecoptera (Mysore et al. 2011; Piersanti et al. 2020, 2024).

In this context, the present study investigates the antennae and brain of the immature stages of the caddisfly *Hydropsyche pellucidula* (Andersen and Klubnes 1834), using scanning and transmission electron microscopy for the antennae and fluorescence microscopy for the brain. We provide (1) a description of larval antennal sensilla across different developmental stages and (2) a preliminary account of brain anatomy in larvae and mature pupae.

The aim is to address the following questions: (1) Which is the sensory equipment of larval antennae? (2) How is the assortment of antennal sensilla reflected in changes in brain structure during development? (3) How do these changes compare with those in Lepidoptera? (4) Is there a relationship between these features and the aquatic lifestyle of caddisfly larvae?

For comparison with adult caddisflies, we refer to the antennal sensilla described by Abu Diiak et al. (2021) in the same species and to the brain anatomy reported for *R. nubila* by Yuvaraj (2017) and Adden (2026).

This research sheds light on the transition from predominant mechanosensory systems in aquatic caddisfly larvae to important chemosensory systems in adults, deepening knowledge of the poorly investigated sensory abilities of Trichoptera (Crespo 2011). In addition, it enhances our understanding of insect sensory biology from an evolutionary perspective, since caddisflies are one of the most important orders of aquatic insects (Crespo 2011) and the closest relatives of Lepidoptera, a key model system in insect sensory biology.

## Materials and methods

### Insects

Larvae of *H. pellucidula* (Trichoptera, Hydropsychidae) were collected on Nera River (Vallo di Nera, Perugia) in January–August 2024. The species was identified using available keys for Trichoptera (Moretti 1983). Because the development in Trichoptera encompasses 5 larval instars distinguishable from morphometric parameters (Andersen and Klubnes 1983; Waringer 2021), the maximum head width and length, together with the total length of the specimens, were measured to attribute the correct larval instar to specimens.

In the laboratory, insects were kept in polystyrene refrigerated boxes with moist paper and no food at 17 ± 2 °C until they were used. Only 4th and 5th instar larvae were used for the research because of technical difficulties associated with the small dimensions of earlier stages and, more importantly, because smaller larvae are short-lived, representing only a small fraction of the immature stage. Indeed, *H. pellucidula* is a univoltine filter-feeding species and during almost the entire year larvae of 3rd to 5th instars appear in the stream with their intricate nets (Poepperl 2000). The species overwinter as 3rd to 5th instar larvae (Andersen and Klubnes 1983) and 5th instar larvae are always present in permanent streams in southern Europe (Muñoz 2003). Some larvae pupated in the laboratory. These pupae were maintained in controlled conditions in water (17 ± 2 °C and absence of light) for approximately 2 weeks. We used them as mature pupae (pharate imago) for neuroanatomical observations.

### Scanning electron microscopy

To describe the antennae and their sensilla during development, head capsules were observed under scanning electron microscopy (SEM). Head of larvae belonging to the 4th and 5th stages were dissected from anaesthetised specimens (10 larvae for each stage) and fixed for 12 h in 2.5% glutaraldehyde in cacodylate buffer (Electron Microscopy Sciences) pH 7.2. Fixed heads were repeatedly rinsed in the same buffer and then dehydrated using ascending ethanol gradients (20%, 50%, 70%, 80%, 95%, 100%), followed by drying in an oven at 40 °C for 3 days. The morphology of the samples was analysed by field emission scanning electron microscopy FE SEM LEO 1525 (ZEISS, Oberkochen, Germany). The specimens were deposited on aluminium supports using adhesive tape. Before the analysis, the samples were covered with a thin layer of chromium (8 nm) using a Q150 T ES (Quorum, Laughton, UK) sputter coater for 25 s. Observations were carried out using an In-lens detector at 5 kV. The number and distribution of the antennal sensilla were observed. Sensilla were named as in previous publications on the larval antennae of other species of caddisflies (Akent’eva, 2012; Friedrich et al. 2015).

### Transmission electron microscopy

To describe the internal structure of the antennal sensilla, 6 antennae were observed under transmission electron microscopy (TEM). The larval head around the mandibular base was dissected and fixed for 3 h in 2.5% glutaraldehyde in cacodylate buffer (Electron Microscopy Sciences, Hatfield, England), pH 7.2. The fixed material was repeatedly rinsed in sodium cacodylate buffer and post-fixed for 1 h at 4 °C in 1% osmium tetroxide in sodium cacodylate buffer (Electron Microscopy Sciences). The samples were then repeatedly washed in the same buffer, dehydrated in ascending ethanol concentrations and finally embedded in an Epon-Araldite resin mixture (Sigma-Aldrich). Afterwards, ultra-thin sections were cut using a Leica EM UC6 ultramicrotome (Leica Microsystem GmbH, Wetzlar, Germany), collected on formvar-coated copper grids, and examined using a TEM Philips EM 208 (Philips, Eindhoven, the Netherlands). The largest seta located ventrally around the mandibular base was used as a visible reference to cut transverse ultra-thin sections of the antenna.

### Neuroanatomy

The brain structure of *H. pellucidula* larvae and mature pupae was visualized with a monoclonal antibody against the *Drosophila* vesicle-associated protein synapsin 1 (SYNORF1, Developmental Studies Hybridoma Bank, University of Iowa, Iowa City, IA, USA). The insect heads were opened with a small blade, and immersed in 4% EM-grade formaldehyde solution (Fisher Scientific, Illkirch, France) in PBS for 1 to 3 h. Brains were subsequently dissected in PBS and post-fixed overnight in 4% EM-grade formaldehyde. All brains were submitted to anti-synapsin immunostaining as described earlier (Piersanti et al. 2020). Briefly, after rinsing and pre-incubation with normal goat serum (NGS), brains were incubated with the anti-synapsin 1 antibody (1:25 in PBS with 0.5% Triton X and 2% NGS for 3 to 5 days). Then incubation with the secondary antibody for 2 to 3 days was done (1:250 in PBS with 1% NGS; Alexa-Fluor-488-conjugated anti-mouse IgG; Invitrogen, Thermo Fisher Scientific, France). After rinsing and dehydration, brains were immersed in methyl salicylate for clarification and subsequently mounted in Permount (Fisher Scientific, Illkirch, France) on aluminium slides between two cover glasses.

Images were acquired with a PlanFluor objective (10 ×/NA 0.3) on a Nikon A1 confocal microscope with excitation wavelengths of 488 and 561 nm. Confocal image stacks were recorded at 1024 × 1024 pixels with a 4 × frame average and a step size of 2 or 3 µm. Stacks were transferred to Fiji software (ImageJ, version 2.0.0, National Institutes of Health, Bethesda, MD, USA) and individual sections were selected for visualization. TIFF files of stacks were saved and transferred to Amira 3.1.1 software (Visualization Sciences Group, Mérignac, France) for 3D reconstructions. 3D models were employed to visualize and qualitatively characterize brain morphology. We obtained six reasonably well-stained preparations from larval stages (4th and 5th instar larvae) and five preparations from mature pupae. However, several of these preparations were not complete due to damage during dissection. Nevertheless, we would like to include these preliminary results here, because of the difficulties to maintain these insects in laboratory conditions and because larval and pupal brains have not been described at all previously.

## Results

### Larvae: antennal morphology and sensilla

**Fig. 1:**
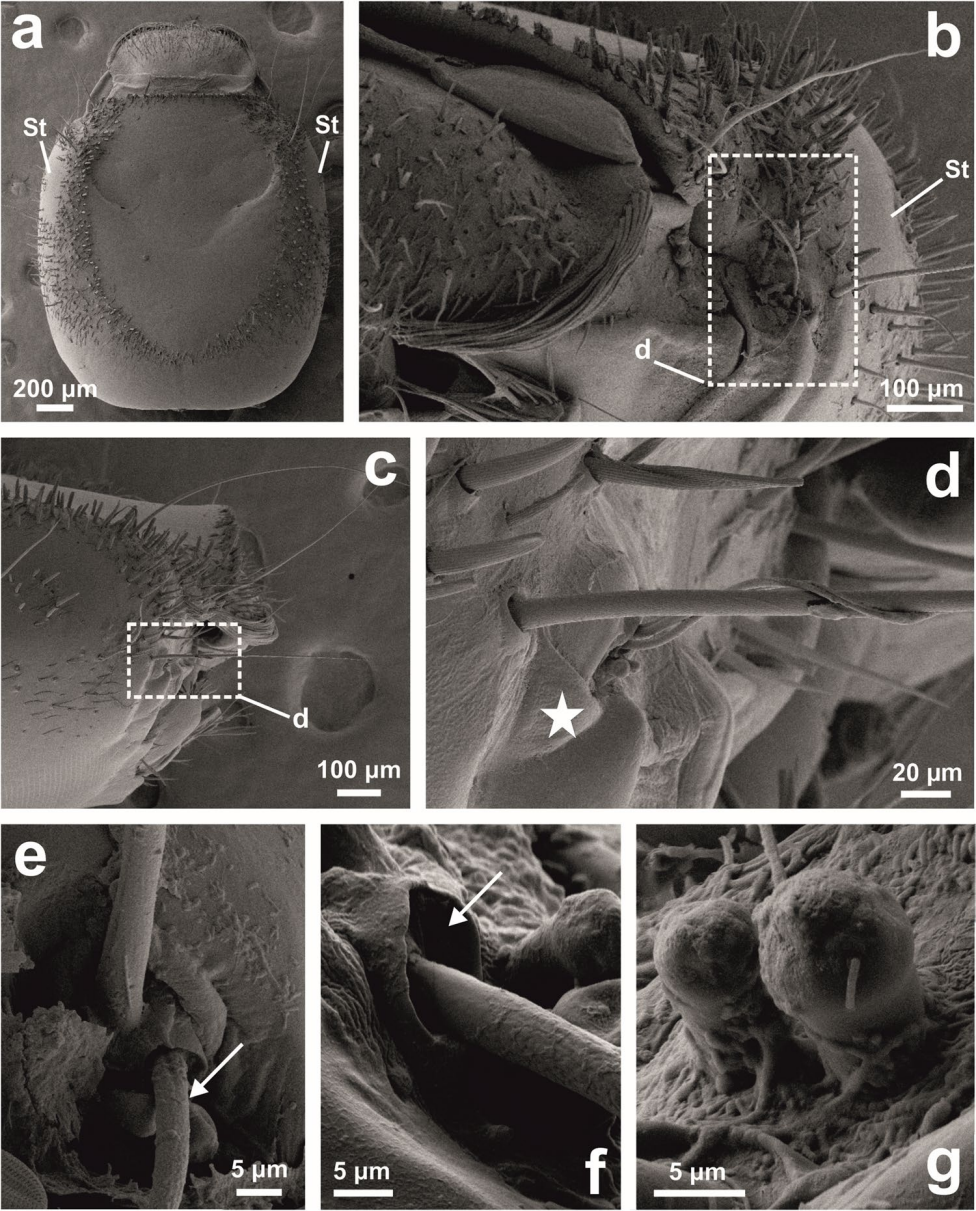
Antennal morphology of *Hydropsyche pellucidula* larvae observed with scanning electron microscopy (SEM). **a** Head of a 4th instar larva in a dorsal view, antennae are not visible. **b** Frontal view of the head area around the mandibular base, note the antenna in the dashed rectangle (d).** c** Lateral view of the head area around the mandibular base, note the antenna in the dashed rectangle (d).** d** Antenna emerging from the torulus (star). **e** Antenna of a 5th instar larva, clearly bearing two trichoid sensilla (arrow). **f** Base of one trichoid sensillum with the visible socket (arrow). **g** Two large sensilla basiconica at the base of the antenna, no pores are visible on the cuticle. St, stemmata

Larval antennae of *H. pellucidula* are not visible on the dorsal view of the head (Fig. 1a) but they are recognisable from the frontal (Fig. 1b) and lateral side (Fig. 1c) of the head Each antenna is unsegmented, highly reduced and is located ventro-laterally between the stemmata and the mandibular base (Fig. 1b–d). The antennal torulus appears as a bulge with a diameter of approximately 50 µm at its base (Fig. 1d). This bulge bears two thin, articulated hairs (Fig. 1b, d, e), which are approximately 5 µm wide at the base and 150–180 µm long with a distinct socket (Fig. 1d–f). Additionally, there are two large sensilla basiconica, without sockets or visible pores (Fig. 1d, g).

**Fig. 2:**
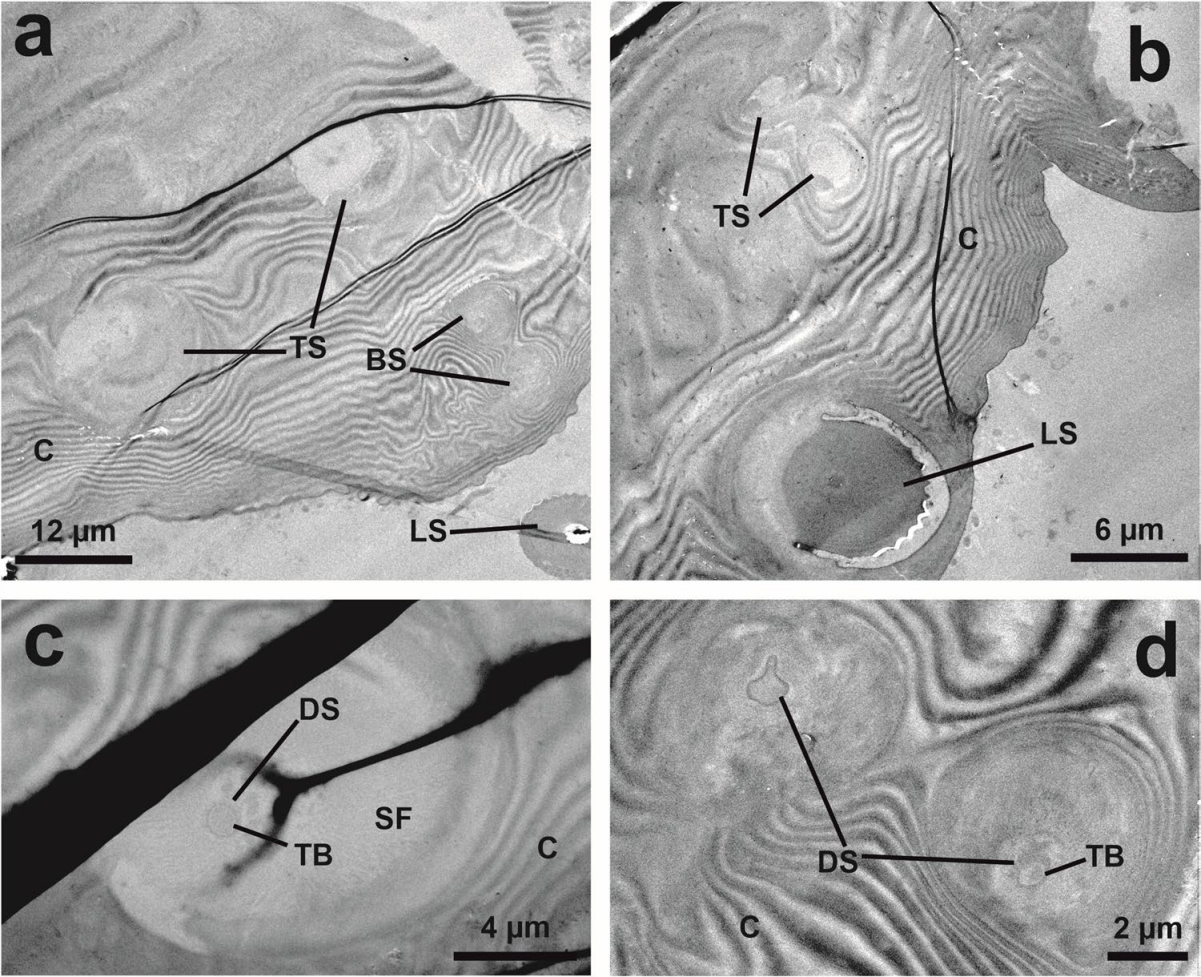
Ultra-thin sections of the antenna of *Hydropsyche pellucidula* larvae observed with transmission electron microscopy (TEM). **a, b** Serial ultra-thin sections (from distal a, to proximal, b) of the antenna, cut using one large seta (LS) as a visible reference. Note that each trichoid sensillum (TS) and each basiconic sensillum (BS) is innervated by a single sensory neuron. **c, d** Detailed view of the trichoid (c) and basiconic (d) sensilla, with dendrites surrounded by the dendritic sheath (DS) forming a prominent tubular body (TB). C, cuticle; SF suspension fibres

Ultra-thin sections of the antenna (Fig. 2) reveal that both trichoid and basiconic sensilla are innervated by a single neuron (Fig. 2a), surrounded by a distinct dendritic sheath (Fig. 2c, d). These neurons are characterised by a prominent tubular body connected to the cuticle by suspension fibres (Fig. 2c, d). The large seta, used as a visible reference for cutting transverse ultra-thin sections of the antenna, is innervated by a single neuron that terminates at the base of the shaft, while the lumen of the seta remains empty (Fig. 2a, b).

No relevant differences have been noticed in 4th and 5th instar larvae.

### Larvae and pupae: brain morphology

We obtained only few intact larval brain preparations due to difficulties with their dissection. In addition, neuropil structures were not very well defined in the optical sections and were therefore difficult to describe. Pupal brains, on the other hand, were easy to dissect, and neuropil structures could easily be seen in optical sections.

**Fig. 3:**
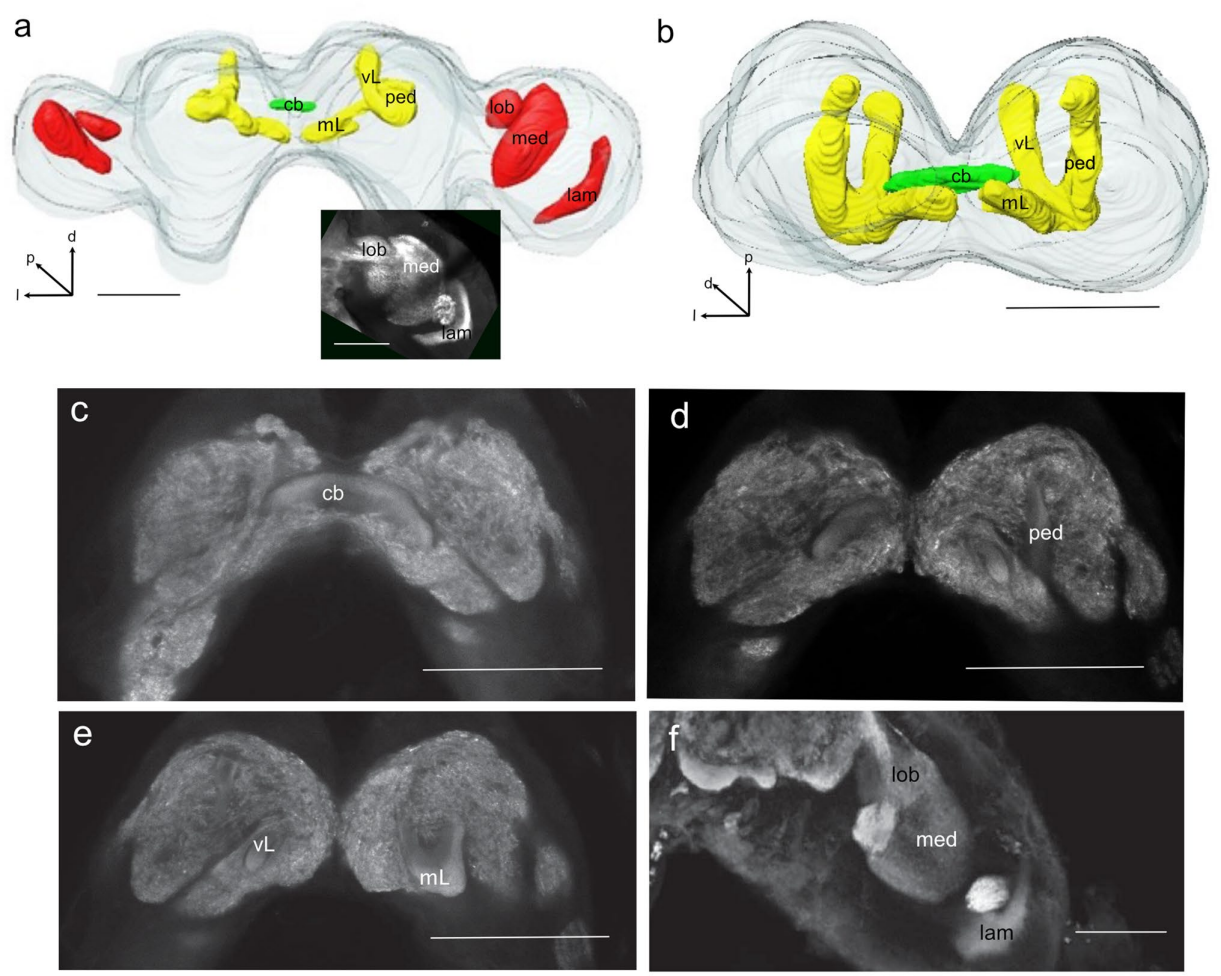
Morphology of the larval *Hydropsyche pellucidula* brain. **a, b** Three-dimensional reconstructions of the complete brain (a) **inset** maximum intensity z-projection of the optic lobes, and a brain without optic lobes (b). **c–e** Single frontal optical sections through another brain, showing the central body (cb) and the peduncle (ped) and the vertical (vL) and medial (mL) lobe of the mushroom bodies). **f** Maximum intensity z-projection of oblique sections through a different larval brain, showing slightly elongated optic lobes.d dorsal, l lateral, lam lamina, lob lobula, med medulla, p posterior. Scale bars 100 µm

In the few larval brain preparations we obtained, we could clearly discern a small central body, but columnar elements could not be clearly seen (Fig. 3a, b, c). Mushroom bodies consisted of a peduncle and ventral and medial lobes, whereas calyces could not be identified (Fig. 3a, b, d, e). Small, slightly elongated optic lobes, appeared to consist of lamina, medulla and lobula (Fig. 3 inset, f). We did not identify antennal lobes and antennal nerves in any of the preparations.

**Fig. 4:**
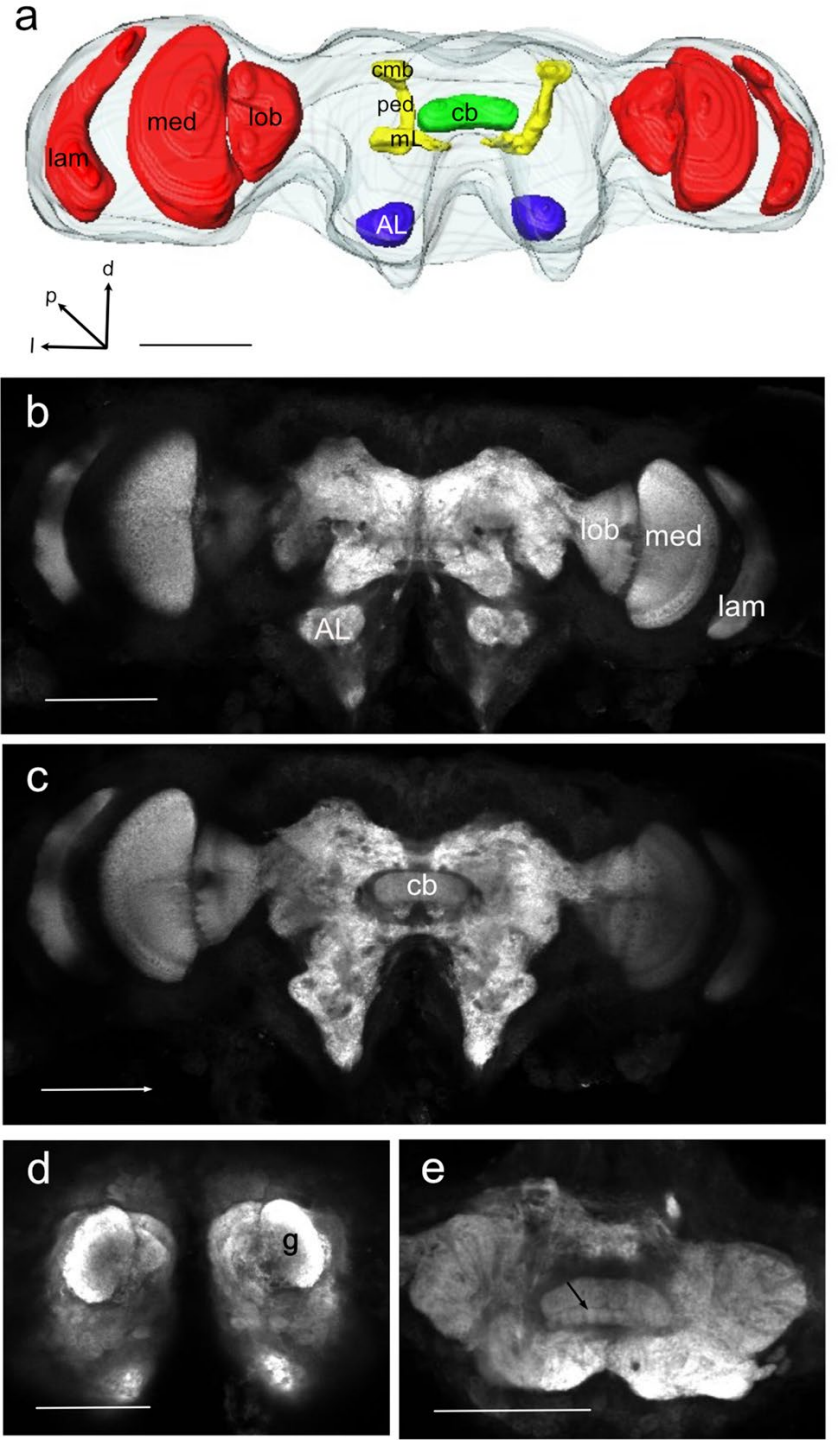
Morphology of the pupal *Hydropsyche pellucidula* brain.** a** Three-dimensional reconstruction of the complete brain. **b, c** Single optical sections through the brain reconstructed in a, showing in b the optic lobes, lamina (lam), medulla (med) and lobula (lob), and the antennal lobes (AL) and in c the central body (cb). **d** Single optical section through the antennal lobes with glomerular structures (g) in a different brain. **e** Single optical section through another brain, showing the columnar structure of the central body (arrow). ped, peduncle; mL, medial lobe and cmb, calyces of the mushroom bodies. Scale bars 100 µm

The brain of *H. pellucidula* pupae strongly resembles the adult brain structure, as described for *Rhyacophila nubila* by Adden (2026). The protocerebrum contains prominent and compact optic lobes with lamina, medulla and lobula (Fig. 4a, b, c) and a central body with a vague fan-shaped structure (Fig. 4e) and mushroom bodies with a peduncle and small calyces, as well as a medial lobe (Fig. 4a). The deutocerebrum contains well-structured antennal lobes with few, but relatively large glomeruli (Fig. 4b, d).

## Discussion

### Antennal morphology and sensilla

The larval antenna of *H. pellucidula* is extremely reduced, likewise in all Trichoptera species, particularly compared to the long antennae of the adults (Melnitsky and Ivanov 2011; Abu Diiak et al. 2021). In all larval stages, the antenna consists of a single unsegmented bulge that bears the same four sensilla.

Two long and thin trichoid sensilla emerge from the middle of the antenna and are characterized by a visible socket and a joint membrane. They are innervated by a single sensory neuron, with a dendrite characterised by a well-developed tubular body, structural characteristics typical of mechanoreceptors (see review in Keil 1997). Similar trichoid sensilla have been reported in the antennae of other caddisfly larvae (see review in Crespo 2011), where they are hypothesized to aid in locomotion and feeding activities, as observed also in some caterpillars (Tozer 1982).

Two club-like sensilla are located at the base of the antenna and lack visible sockets or pores on their cuticle. We named these sensilla basiconica, in agreement with previous publications on other species of caddisflies (Akent’eva, 2012; Friedrich et al. 2015). Similar sensilla have been described in *Rhyacophila* larvae, where no functional hypothesis has yet been proposed (Friedrich et al. 2015). They are likely a groundplan feature of caddisfly larval antennae (Friedrich et al. 2015; Crespo 2011; Frania and Wiggins 1997). In *H. pellucidula*, the internal structure of sensilla basiconica suggests a mechanoreceptive role; indeed, they are innervated by a single dendrite, surrounded by a dendrite sheath and equipped with a developed tubular body (see review in Keil 1997). The small size, as well as their shape and position as small domes at the base of the antenna, combined with the absence of an articulated socket and the presence of an irregular sensillar cuticle, suggests a potential function as stretch and pressure receptors. Because of their shape, they are probably not capable of perceiving water currents, as trichoid sensilla probably are.

The adult antenna in caddisflies of the family Hydropsychidae bears eleven types of mechanoreceptors and chemoreceptors on the flagellum, including those that are typical of most caddisfly antennae (Abu Diiak et al. 2021). Six of these types (long trichoid, curved trichoid, pseudoplacoid, chaetoid, coronary, and styloconic sensilla) are shared with many Lepidoptera (Valuyskiy et al. 2020). Additionally, three unique types are found only within the caddisflies of the family Hydropsychidae: T-shaped pseudoplacoid sensilla, ribbed pseudoplacoid sensilla, and thick chaetoid sensilla (Abu Diiak et al. 2021). None of the adult sensilla resembles the larval ones, including the long trichoid sensilla, which in adults are more numerous and much thicker (Abu Diiak et al. 2021). The marked difference between larval and adult sensilla in caddisflies reflects their distinct ecological roles. In adults, antennae play a crucial role in pheromone communication (Löfstedt et al. 1994, 2008; Jewett et al. 1996; Bergmann et al. 2001; Melnitsky and Ivanov 2011), whereas in larvae, chemoreception seems to be reduced, sometimes reported as related to predatory avoidance (Gall and Brodie 2009; Okano et al. 2017) and primarily mediated by mouthparts (Spanhoff et al. 2005; Crespo 2011).

The antenna of larval *H. pellucidula* is very similar to that of *Rhyacophila fasciata* (Friedrich et al. 2015) but differs significantly from those described in *Limnephilus centralis* (Akent’eva, 2012), which is equipped with olfactory and gustatory sensilla and has a cylindrical rather than a bulging form. According to Friedrich et al. (2015), the reduced antenna of *H. pellucidula* does not represent the “primitive type” in Trichoptera but is more likely the result of secondary reduction. This interpretation is consistent with the broader phylogenetic framework proposed by Frania and Wiggins (1997), in which larval antennal morphology in Trichoptera is considered highly reduced and conservative. In their analysis, the larval antenna is treated as a simplified, non-articulated structure, and variation in its morphology is interpreted primarily in terms of reduction rather than retention of ancestral states. Consequently, the condition observed in *H. pellucidula* is unlikely to represent a plesiomorphic condition, but rather reflects a derived state within a general trend toward antennal reduction in the order. This supports the view that similarities in antennal structure among taxa should be interpreted with caution, as they may result from convergent or parallel reduction rather than from shared ancestry in Trichoptera.

Antennal differences and similarities within the order may offer valuable insights into the phylogenetic relationships of caddisflies. Indeed, Hydropsychidae and Limnephilidae are clearly distant taxa, with the former belonging to Annullipalpia and the latter to Integripalpia (Thomas et al. 2020), while the phylogenetic position of Rhyacophilidae is more uncertain and debated. Some authors place them within Annullipalpia, others within Integripalpia, and some in a third taxon called Spicipalpia (Thomas et al. 2020). In this context, larval antennal morphology could potentially enhance the character set used to improve our understanding of caddisfly taxonomy, as has been proposed for adult antennae (Abu Diiak et al. 2021).

### Brain morphology

The strong reduction of the larval antenna and the lack of clearly identifiable olfactory sensilla, together with the clear presence of mechanoreceptors, agree with our observation of the absence of the antennal lobe in the larval brain. Also, the calyces of the mushroom bodies, identified in many insects as the main secondary processing centre for olfactory information as well as a centre of learning and memory (Strausfeld et al. 2009), seem to be highly reduced in our larval brains. Our data reveal that the calyces of the mushroom bodies appear in the pupal brain, similar to the glomerular antennal lobes, likely preparing the structures needed to process incoming olfactory information from adults' well-developed antennae.

This situation seems to differ from that of other aquatic insects in which the antennal pathway from sensilla to the brain has been investigated during development, such as Odonata (Piersanti et al. 2020) and Plecoptera (Piersanti et al. 2024). These ancient hemimetabolous insects possess different olfactory sensilla on the antennae at nymphal and adult stages, but very similar neuropils in the brain. Thus, the same brain structures are likely to process information perceived in water and in air (Piersanti et al. 2020; 2024), despite the substantial differences in chemosensory inputs (Crespo 2011). This aligns with developmental plasticity, which helps maintain functionality throughout ontogenesis when the absence of a pupal stage precludes metamorphic changes in the nervous system.

What we observed in the antennae and brain of *H. pellucidula* is similar to the results obtained in the mosquito *Aedes aegypti*, in the only study, to our knowledge, on the development of the brain in holometabolous insects with aquatic larval stages and flying adults (Mysore et al. 2011). In this case, the authors describe the complete transformation and marked growth in size of the brain neuropils from stage 4 larvae to adult mosquitoes, passing through 24-h pupae. In particular, the complex neuropils characteristic of the adult brain, such as the antennal lobe, central complex, and the three optic lobe neuropils, are formed during the pupal stage, similarly to *Drosophila* (Mysore et al. 2011). Notwithstanding the different ecology of caddisfly larvae compared to mosquitoes, which inhabit standing waters and filter decaying matter, rather than living in running waters and capturing food using specialised nets, caddisflies are also holometabolous insects. Thus, they undergo a pupal stage during which brain morphology can change dramatically. In our brain preparations, we observed markedly reduced neuropils in the larva that transform into the well-developed neuropils seen in the mature pupa, which are essentially identical to those described in adults (Yuvaraj et al. 2017; Adden 2026).

In Lepidoptera, all major neuropils of the adult central brain, such as glomerular antennal lobes or mushroom bodies with large calyces, are also present in the last larval stage, without major differences between families or between nocturnal and diurnal species (Nordlander and Edwards 1968; Homberg and Hildebrand 1994; Huetteroth et al. 2010; Sehadova et al. 2023). The difference suggested by our preliminary data, between brain development in Trichoptera and Lepidoptera, could be related to the aquatic lifestyle of caddisfly larvae. Indeed, the density of water allows vibrations to be transmitted much farther than in the atmosphere, with the consequence that many aquatic organisms, including insects, rely heavily on mechanoreception to detect the environment (Rebora et al. 2019). Unlike adults, lepidopteran caterpillars typically do not use pheromones, but they still perceive odorants in the terrestrial environment to orient their behaviours, such as host plant selection and predator avoidance (Wang et al. 2025). By contrast, larval caddisflies probably mainly rely on mechanoreception (Sakhuja et al. 1983) to remain in the right portion of the stream and to feed on small moving prey or particles trapped by their nets. This is probably why the larvae of *H. pellucidula* show clear mechanoreceptors on their antennae, and why the evolutionary trend in the larval antenna of Trichoptera is toward reduction of chemoreception (Friedrich et al. 2015). The presence of olfactory processing circuitry in Crustacea and basal Insecta (Mißbach et al. 2011; Schachtner et al. 2005), together with neuroanatomical investigations in Entognatha such as Collembola (Kollmann et al. 2011) and in the myriapod *Scutigera coleoptrata* (Sombke et al. 2011), all showing glomerular ALs, suggests that the absence of well-developed olfactory neuropils may be a derived condition even in other aquatic insects such as Odonata, notwithstanding their basal phylogenetic position among winged insects (Farris 2005; Strausfeld et al. 2009; Rebora et al. 2013). Further morphological and neuroanatomical investigations on caddisfly species such as *Limnephilus centralis*, which are reported to possess chemoreceptors on their small larval antennae (Akent’eva, 2012), could better clarify this aspect and confirm our hypothesis.

## Conclusions

Our results suggest that caddisflies undergo dramatic changes in their antennal sensory equipment and brain sensory pathways during development. In particular, larval antennae show morphological evidence for mechanoreception and a lack of clear olfactory sensilla, which are well developed in adult antennae. This agrees with our preliminary evidence in brain morphology, which suggests the absence of the main olfactory centres in larvae and the presence of glomerular antennal lobes and well-developed calyces in the mushroom bodies of pupae and adults. This seems to differ from what occurs in Lepidoptera and may be related to a reduction in larval antennal sensitivity in caddisflies, despite their basal phylogenetic position relative to butterflies and moths. Further investigations are needed to clarify this interesting aspect of the evolutionary processes in insect olfaction and confirm these hypotheses.

## Data Availability

No datasets were generated or analysed during the current study.
